# Pulsed Resource Events Mediate Fluctuations in Wild Boar (*Sus scrofa*) Bag Records in Central Europe

**DOI:** 10.1002/ece3.73874

**Published:** 2026-07-01

**Authors:** Robert Hagen, Christian Fiderer, Janosch Arnold, Toralf Bauch, Andreas Elliger

**Affiliations:** ^1^ Agricultural Center Baden‐Württemberg Wildlife Research Unit Aulendorf Germany; ^2^ Forest Research Institute Baden‐Württemberg FVA‐Wildlife Institute Freiburg Germany

**Keywords:** *Fagus sylvatica*, mast seeding, population fluctuations, *Quercus petraea*, *Quercus robur*

## Abstract

Climate change in Europe is leading not only to an increase in temperature but also to a higher frequency of extreme weather events. Knowledge of the cascading effects of climate change along food chains, including mast seeding and the population fluctuations of seed consumers, is therefore essential. In this study, the spatio‐temporal patterns of wild boar ( *Sus scrofa* ) bag records at the European level (2000–2023, 16 countries) and across 13 federal states in Germany (2000–2023) were investigated. Data from the federal state of Baden‐Württemberg (southern Germany) were then analyzed in detail to understand the relationship between mast seeding by European beech ( *Fagus sylvatica* ), sessile oak ( *Quercus petraea* ), and pedunculate oak ( *Quercus robur* ), the number of wild boar shot, the number of wild boar‐vehicle collisions, and the temperature conditions during April and May. We detected bi‐annual fluctuations in wild boar bag records that were most pronounced and spatially synchronized over large parts of Europe between 2007 and 2012 and between 2017 and 2020. In Baden‐Württemberg, warm spring conditions during April and May were associated with intensive mast seeding by both oak species ( *Q. petraea* ; *Q. robur* ) and by beech ( *F. sylvatica* ). The amount of energy available for wild boar due to mast seeding by these three tree species was counterintuitively negatively associated with the number of wild boar shot (2000–2022) and with the number of vehicle collisions involving wild boar (2000–2022). Between 2000 and 2022, both time series peaked during years without mast seeding. Our results suggest a cascading effect of springtime temperature conditions, via oak and beech mast seeding, on the wild boar populations of Central Europe.

## Graphical abstract

Warm spring conditions during April and May were associated with mast seeding by both oak species ( *Q. petraea* , *Q. robur* ) and beech ( *F. sylvatica* ). The amount of energy available for wild boar in southern Germany due to mast seeding by these three tree species was negatively associated with the number of wild boar shot and with the number of vehicle collisions involving wild boar.

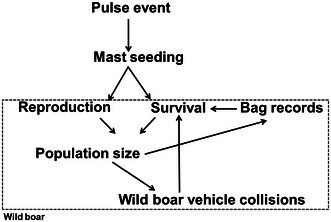

## 1 Introduction

The number of wild boar in Europe and Northern Asia has increased since the 1960s (Markov et al. 2022; Massei et al. 2015), accompanied by an expansion of their range in Northern Eurasia (Bergqvist et al. 2024; Markov et al. 2022). Wild boar bag records in many European countries have also increased (Massei et al. 2015), albeit with variations in time and space as well as between and within European countries. Thus, following a sharp decline in wild boar bag records in almost all European countries in 2006 (Massei et al. 2015), the yearly fluctuations in Central Europe between 2007 and 2013 have been largely similar (Massei et al. 2015). This synchronous temporal pattern in ungulate bag records on a continental scale points to factors affecting population size, as suggested for roe deer ( *Capreolus capreolus* ) (Hagen et al. 2017). Large‐scale population fluctuations are induced by climatic variations, the availability of habitat and resources, and species‐specific interactions (Elton 1924; Myers and Worm 2003; Nicholson 1933). Fluctuations characterized by an irregular temporal pattern, without a fixed oscillation period, are called quasi‐cycles (Hagen et al. 2017). Quasi‐cycles with a main oscillation period of 2 years (bi‐annual fluctuations) point to a high frequency of forcing events that affect vital rates (Hagen et al. 2017). For wild boar, a likely forcing event on a continental scale is a resource pulse (seed production of trees) that affects the number of individuals able to survive and reproduce during winter (Touzot et al. 2023, 2020).

The synchronous production of large seed crops by plants is a feature of many terrestrial ecosystems (Bogdziewicz et al. 2024; Pesendorfer et al. 2021). These pulsed food resource events are common in woody and wind‐pollinated species (Herrera et al. 1998), where they are referred to as mast seeding. Synchronous mast seeding events by a specific tree species are characterized by an annual seed production that exceeds the average seed production over a regional to continental spatial scale (Ascoli et al. 2017; Bogdziewicz et al. 2023). Recent decades have seen both an increase in the inter‐annual variation in seed production of many tree species (Ascoli et al. 2017; Pearse et al. 2017) and a higher frequency of mast seeding events in the northern hemisphere, as described for beech ( *Fagus sylvatica* ) in Europe (Müller‐Haubold et al. 2015) and for oak (*Quercus crispula*) in Asia between 1980 and 2017 (Shibata et al. 2020).

Among the factors that result in mast seeding are resource availability and fitness benefits for tree species (Bogdziewicz et al. 2024; Dale et al. 2021; Koenig 2021) and weather cues, such as air temperature during the flowering period (Abe et al. 2016; Vacchiano et al. 2017; Caignard et al. 2017). However, their interaction is poorly understood (Pearse et al. 2016; Shibata et al. 2020), and the impact of climate change on tree‐specific mast seeding is complex (Bogdziewicz et al. 2024). Several studies have shown that mast seeding alters the population dynamics of non‐insect seed consumers, including rodents, birds (Köhnke et al. 2020; Ostfeld et al. 1996; Andreassen et al. 2021), and ungulates such as wild boar (Gamelon et al. 2021, 2019; Touzot et al. 2023, 2020). A periodicity of mast seeding events is therefore likely to induce population fluctuations in those species.

The objective of this study was to examine the spatial dimension underlying the bi‐annual fluctuations in wild boar bag records in Europe since 1995/2000 and their relationship to mast seeding events. We therefore investigated the spatio‐temporal synchrony in wild boar bag records in Germany and Europe since 2000 and then analyzed the data for the federal state of Baden‐Württemberg with respect to: (i) mast seeding by *Fagus sylvatica* , *Quercus petraea* , and *Quercus robur* (1995–2022); (ii) the number of wild boar shot (2000–2022); (iii) the number of wild boar‐vehicle collisions (2000–2022); and (iv) the temperature during April and May—corresponding to the period of wind pollination of oak and beech in southern Germany (1995 and 2022).

## 2 Data and Methods

### 2.1 Bag Records

Data on the official number of wild boar shot and the number of wild boar involved in vehicle collisions in Baden‐Württemberg, as recorded by local authorities, are sent annually to the Wildlife Research Unit of Baden‐Württemberg (Table S2). For the other German federal states (excluding the city‐states Berlin, Hamburg, and Bremen), data on wild boar bag records between 2000 and 2023 were obtained from the German Hunting Association (DJV, Deutscher Jagdverband, Table S3). Annual bag records in European countries were obtained from numerous institutions (Table 1). Time series analyses for bag records focused on time series over at least 20 years, beginning in 2000 (Table 1; Table S4).

**TABLE 1:** Availability of annual wild boar bag records in Europe.

Country	Period	Source
Albania	—	—
**Austria**	1982–2023	1982: Massei et al. (2015) **1983–2023: Statistik Austria** https://www.statistik.at/datenbanken/statcube‐statistische‐datenbank
Belgium	1982–2013	Massei et al. (2015)
Belarus	—	—
Bosnia and Herzegovina	—	—
Bulgaria	1960–1979	Briedermann (1986)
**Croatia**	1990–2022	1990–1993: Massei et al. (2015); **1994–2022: Croatian Bureau of Statistics** https://web.dzs.hr/PxWeb/pxweb/en/
**Czech Republic**	1982–2023	1982–1999: Massei et al. (2015); **2000–2023: Czech Statistical Office** https://csu.gov.cz/
Denmark	2011–2022 (first record in 2011)	Aarhus University https://fauna.au.dk/en/hunting‐and‐game‐management/bag‐statistics/
**Estonia**	1992–2023	**Tartu University (Harri Valdmann)**
Italy	1985–2011	Massei et al. (2015)
Finland	2008–2023 (first record in 2008)	Luke—Natural Resources Institute Finland https://statdb.luke.fi/PxWeb/pxweb/en
**France**	1973–2023	**Office Francais de la Biodiversité** https://www.ofb.gouv.fr/en
**Germany**	1958–2023	**Deutscher Jagdverband** https://www.jagdverband.de/
Great Britain	2008–2021 (first record in 2008)	The Boaring Truth https://theboaringtruth.org/management
Greece	—	—
**Hungary**	1970–2023	1970–1983: Briedermann 1986; 1984–1989: Csányi (1995); **1990–2023: Hungarian Central Statistical Office** https://www.ksh.hu/stadat_files/kor/en/
Kosovo	—	—
**Latvia**	1980–2023	**Valsts meža dienests** https://www.vmd.gov.lv/lv
**Lithuania**	1990–2023	**Linas Balčiauskas (State Scientific Research Institute Nature Research Centre, Lithuania)**
Luxembourg	1982–2013	Massei et al. (2015)
Moldova	—	—
Montenegro	—	—
Netherlands	—	—
North Macedonia	—	—
Norway	2014–2022 (first record in 2014)	Statistics Norway https://www.ssb.no/en/statbank/table/07514/
**Poland**	1970–2023 (without 1979 and 2014)	1970–1982: Briedermann (1986) **1983–2012:** Massei et al. (2015) **2013–2023: Statistical Yearbook of Forestry 2018, 2019, 2020, 2022, 2024**
**Portugal**	1989–2021	Carvalho et al. (2024)
Romania	—	—
Serbia	1982–2023 (since 1995 only every second year)	1982–2003: Massei et al. (2015); 2015–2023: Statistical Yearbook 2015, 2017, 2019, 2021, 2023, 2024
**Slovak Republic**	1968–2023	**Statistical Office of the Slovak Republic** https://datacube.statistics.sk/
**Slovenia**	1982–2023	**1982–2001:** Massei et al. (2015)**; 2002–2023 Statistical Office** https://pxweb.stat.si/SiStatData/pxweb/en/Data/
**Spain**	1990–2022	**1990–2011:** Massei et al. (2015)**; 2012–2022: University of Cordoba (Juan Carranza)**
**Sweden**	1990–2023 (first record in 1990)	**VILTDATA** https://rapport.viltdata.se/statistik/
**Switzerland**	1982–2021	1982–1994: Massei et al. (2015); **1995–2021: Bundesamt für Statistik** https://www.bfs.admin.ch/
Turkey	—	—
Ukraine	1999–2007	Ukrainian Nature Conservation Group https://uncg.org.ua/en/cadastre/monitoring‐of‐numbers‐distribution‐and‐bagging‐of‐game‐species/numbers‐distribution‐and‐bagging‐of‐wild‐boar‐sus‐scrofa/

*Note:* Only those countries with at least 20 years of data between 2000 and 2023 were included in the time series analysis (country names are shown in bold). The temporal pattern of data since 1970 and the countries excluded from the time series analysis are reported in Figures S1–S4.

Standardized time series (mean value = 0, standard deviation [SD] = 1) were calculated for each German federal state (*N* = 13) and each European country (*N* = 16).

### 2.2 Mast Seeding

#### 2.2.1 Step 1—Estimating the Size of Forest Patches

The area of patches with contributing trees per tree species was estimated using official data from the German forest inventory (1987, 2002, 2012; grid size: 4 × 4 km) for the federal state of Baden‐Württemberg (area: ~35,000 km^2^) (Elliger 2025). The area of forest patches (ha) containing trees older than 80 years (oaks) or 60 years (beech), and thus the size of the forest patches that mainly contributed to seed production, was considered (cf. Table S1).

#### 2.2.2 Step 2—Estimating the Biomass

Seed production by *Fagus sylvatica* (FS) ranges between 200 kg and 1000 kg per ha, while seed production by *Quercus robur* (QR) and *Quercus petraea* (QP) ranges between 200 and 1800 kg per ha (Journé et al. 2021). For a pulsed event (high intensity mast seeding), we assumed that seed production by these tree species was relatively high: on average, 825 kg seed per ha (750–900 kg) for FS, 1, 650 kg per ha (1500–1800 kg) for QP and 2350 kg per ha (2200–2500 kg) for QR (Linderoth and Pegel 2010). To obtain an estimate of the dry mass for FG, QR, and QP, the biomass value for the fresh weight was multiplied by 0.5 for QP and QR and 0.85 for FS (Linderoth 2010). This assumption is in line with studies that determined the dry mass of seeds (Obranović et al. 2024—dry weight ~0.75 × fresh weight for FS; Öztürk and Caliskan 2024—dry weight ~0.5 × fresh weight for both oak species).

#### 2.2.3 Step 3—Characterizing the Mast Intensity

Estimates of the intensity of pulsed events were obtained from the *Staatsklenge* [Forestry Service] of the town of Nagold (Elliger 2025). According to Rohmeder (1972), for every tree species, the Staatsklenge Nagold annually registers the intensity of mast seeding: high intensity (“Vollmast”, > 80% of all trees produce seeds), medium intensity (“Halbmast”, 50%–80% of all trees produce seeds), low intensity (“Sprengmast”, 10%–50% of all trees produce seeds), or no mast seeding (“Fehlmast”, < 10% of all trees produce seeds). We multiplied the area (Step 1) by 0.9, 0.65, 0.3, and 0.05, respectively, to obtain estimates for high, medium, low, and no mast seeding (Linderoth 2010, cf. Table S1).

#### 2.2.4 Step 4—Estimating the Available Energy for Wild Boar

The energy available to wild boar individuals (energy for metabolism) was calculated according to the Weender analysis (Linderoth and Pegel 2010). The stomach contents of 20 individuals under conditions of 100% beech seeds (*N* = 10) or 99% oak seeds (*N* = 10) were used to estimate the mean energy for metabolism: 11 Megajoule (MJ) [8–14.4 MJ] per kg dry mass for oak and 13 MJ [10.5–15.6 MJ] per kg dry mass for beech (Linderoth and Pegel 2010).

Finally, the sum of the expected available energy (AE) was calculated for every year:

(1) $${\mathrm{AE}}_{j}\;\left[\mathrm{MJ}\right]={\mathrm{AE}}_{\mathrm{FS},j}+{\mathrm{AE}}_{\mathrm{QP},j}+{\mathrm{AE}}_{\mathrm{QR},j}$$

where FS stands for *Fagus sylvatica* , QP for *Quercus petraea* , and QR for *Quercus robur* , respectively, and *j* is the year. For example, the expected available energy related to beech in 2002 (low mast intensity) can be calculated (cf. Table S1):

(2) $${\mathrm{AE}}_{\mathrm{FS},2002}\;\left[\mathrm{MJ}\right]=196,530\;\left[\mathrm{ha}\right]\times 0.3\times 825\;\left[\mathrm{kg}/\mathrm{ha}\right]\times 0.85\times 13\;\left[\mathrm{MJ}/\mathrm{kg}\right]$$

For visual comparison with the variable temperature during April and May, a standardized time series of AE_FS_, AE_QP_, and AE_QR_ was calculated (mean = 0, SD = 1).

### 2.3 Temperature During April and May

Data on air temperature during April and May were obtained from the German Weather Service (DWD, Table S2). The cumulative effects of temperature on the flowering of beech and oak were taken into account by calculating the sum of the average temperatures in April and May. For a visual comparison with the energy provided by mast seeding events, a standardized time series of the sum of the temperatures during April and May was calculated (mean = 0, SD = 1).

### 2.4 Statistical Analysis

First, a cluster analysis for the wild boar bag records was conducted using the Pearson correlation coefficient as a distance measure (Hagen et al. 2017). The silhouette score was used to determine the optimal number of clusters (Rousseeuw 1987), defined as those > 2. Silhouette scores vary between −1 and 1, where −1 indicates the worst and 1 the best clustering quality.

Statistical associations between (i) the yearly variation in the number of wild boar shot or the number of wild boar‐vehicle collisions and the yearly variation in AE (Equation 1) and (ii) the temperature during April and May and mast seeding by the three tree species were evaluated using simple linear models (Response variable~Temperature; Response variable~Mast seeding). Statistical support for quasi‐cycles was assessed by calculating the empirical autocorrelation function (ACF) for each time series (Pineda‐Krch et al. 2007). We used the cross‐correlation function (CCF) to identify time delays between two distinct time series. All analyses were conducted in R version 4.4.0 (R Core Team, 2024). A *p*‐value < 0.05 was considered to indicate statistical significance.

## 3 Results

### 3.1 Bag Records in Germany (2000–2023)

The silhouette score indicated 3, 5, or 6 different temporal patterns in the bag records of wild boar. The silhouette scores for clusters of 3, 4, 5, 6, and 7 groups were 0.32, 0.18, 0.33, 0.33, and 0.25, respectively. However, for clusters containing ≥ 5 groups, there was always a group consisting of only one federal state. The decision was therefore made to group the federal states according to the results of the 3‐cluster solution, yielding clusters corresponding to northern Germany (3 federal states, Mecklenburg‐Western Pomerania, Lower Saxony, Schleswig‐Holstein), eastern Germany (3 federal states, Brandenburg, Saxony, Saxony‐Anhalt), and western/southern Germany (7 federal states, Bavaria, Baden‐Württemberg, Thuringia, Hesse, Rhineland‐Palatinate, North Rhine‐Westphalia, Saarland).

**FIGURE 1:**
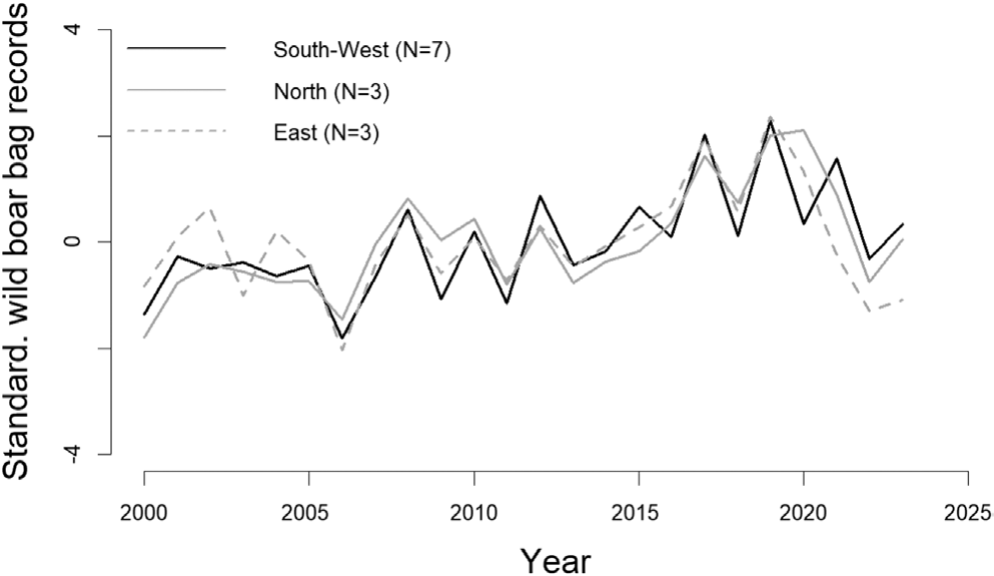
Standardized wild boar bag records for the spatial clusters northern Germany (*N* = 3), eastern Germany (*N* = 3), and southern/western Germany (*N* = 7). Differences in the temporal patterns were associated mainly with the years before 2005 and after 2019.

A comparison of the temporal pattern of these three times series revealed a high degree of similarity even for time series belonging to different clusters (smallest Pearson correlation coefficient equal to 0.75, CI_95_[0.49–0.88] for the standardized time series for the clusters “East” and “South‐West” Figure 1). The time series for Germany's federal states consisted of an absolute minimum number of bag records in 2006, with bi‐annual fluctuations in bag records during the periods 2005–2012 and 2016–2019 (Figure 1). Although the time series were very similar, only those for the federal states in western/southern Germany (7 out of 7) were characterized by significant bi‐annual fluctuations (Figures S5–S7).

### 3.2 Bag Records in Europe (2000–2023)

Time series of wild boar bag records at the country level across Europe partly reflected the effects of African swine fever on wild boar populations (Baltic countries and Poland since 2014, Hungary since 2018, Croatia since 2021; European Food Safety Authority (EFSA) et al. 2025). Consequently, the temporal pattern of wild boar bag records in the Baltic included a sharp decrease in 2014, the year when the virus was confirmed. The silhouette score suggested either four or five different temporal patterns (silhouette score for clusters 3, 4, 5, 6, and 7: 0.4, 0.43, 0.43, 0.35, and 0.28, respectively).

**FIGURE 2:**
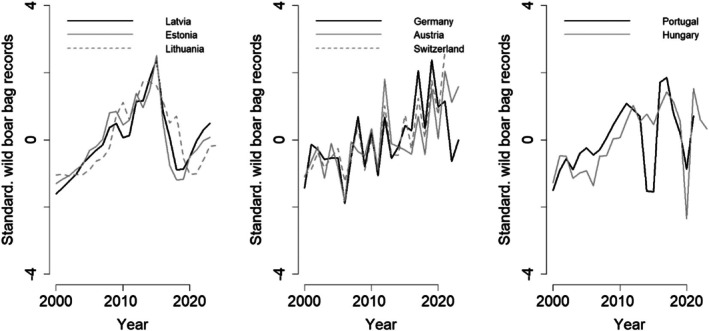
Three different temporal patterns of wild boar bag records in European countries since 2000.

For the temporal pattern attributable to a spatial cluster of neighboring countries in the Baltic region (Estonia, Latvia, and Lithuania), a maximum value in 2013/2014 after which the bag record decreased sharply until 2019/2020 (Figure 2). A second pattern, characterized by significant bi‐annual fluctuations in wild boar bag records, was identified for three Central European countries (Austria, Germany and Switzerland) (Figure 2; Figure S8).

**FIGURE 3:**
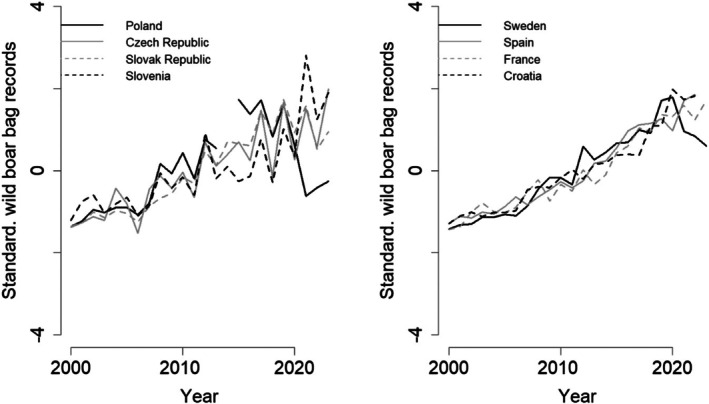
Time series associated with the fourth temporal pattern of wild boar bag records from 2000 to 2020. The time series of this cluster is divided into two groups, one showing more pronounced (left panel) and the other less pronounced (right panel) interannual variations. The time series of Poland contained one missing value for the year 2014.

A third pattern consisted of a period of increasing bag records from 2000 to 2010 and a sharp decrease in 2020. However, the reasons for this decrease differed in the various countries: in Hungary, African swine fever was confirmed beginning in 2017 (European Food Safety Authority (EFSA) et al. 2025); in Portugal, the decrease was due to the COVID‐19 pandemic (Carvalho et al. 2024) (Figure 2). A fourth pattern reflected a largely continuous increase in wild boar bag records, with either more (Czech Republic, Poland, Slovak Republic and Slovenia) or less (Croatia, France, Spain, Sweden) pronounced inter‐annual variations (Figure 3; Figures S9 and S10).

### 3.3 The Effects of Mast Seeding and Spring Temperature in Baden‐Württemberg on Wild Boar Bag Records

**FIGURE 4:**
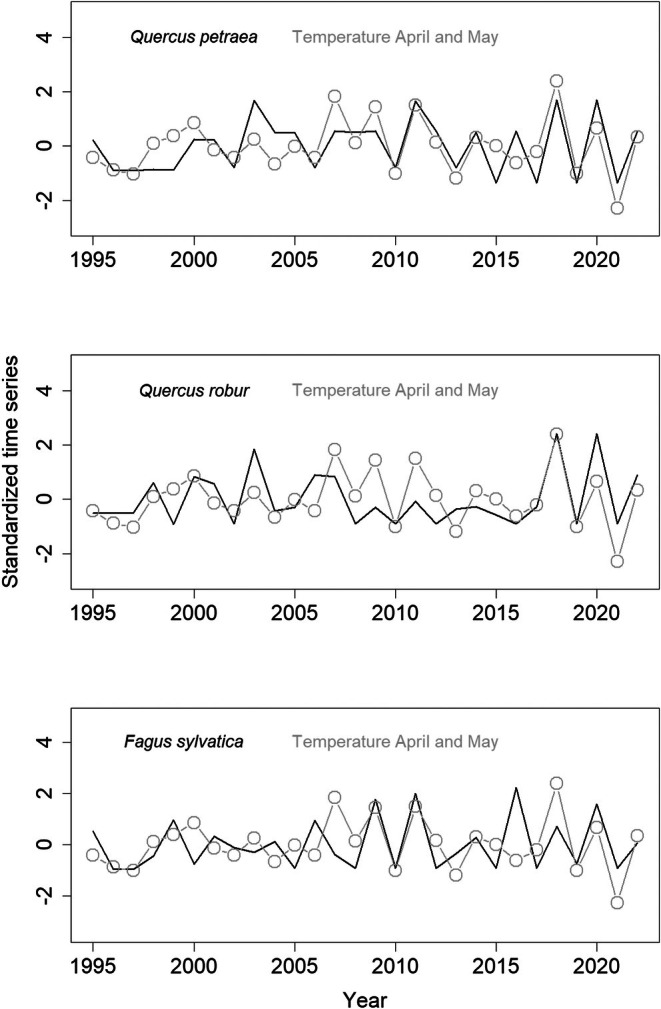
Standardized time series on the available energy of *Quercus petraea* , *Quercus robur* , and *Fagus sylvatica* , and the sum of the average temperatures during April and May in Baden‐Württemberg for the period 1995–2022.

The available energy related to *Q. petraea* , *Q. robur* , and *F. sylvatica* did not increase or decrease significantly after 2000 (*p*‐values of the linear models using year as the covariate were > 0.5) but instead showed strong variations (Figure 4). The mean proportion of AE_FS,*j*_ *to* AE_*j*_ was 0.77 (SD = 0.16), highlighting the importance of *F. sylvatica* for wild boar in Baden‐Württemberg. In the time series of the available energy of *Q. petraea* , bi‐annual fluctuations were detected (Figure S11).

Synchronized mast seeding by all three tree species was observed beginning in 2017 (but as early as 2014 for *F. sylvatica* and *Q. petraea* ). The sum of the average temperatures in April and May did not increase after 1995 (*p*‐value of the covariate *year* in a linear model = 0.83), but increased since 1960, especially during the early 1990s. This increase in spring temperatures can be attributed to the environmental regime shift in Central Europe that began in the late 1980s–early 1990s (Reid et al. 2016). There was no evidence of a bi‐annual oscillation in spring temperature (Figure S12).

In linear models using the available energy of a single tree species as the response variable and temperature (sum of April and May temperatures) as the predictor variable, spring temperature was positively associated with the available energy related to both oak species ( *Q. petraea* : *p* < 0.001, adjusted *R* ^2^ = 0.4; *Q. robur* : *p* = 0.003, adjusted *R* ^2^ = 0.27) and to beech (*p* = 0.01, adjusted *R* ^2^ = 0.18; see Figure 4).

**FIGURE 5:**
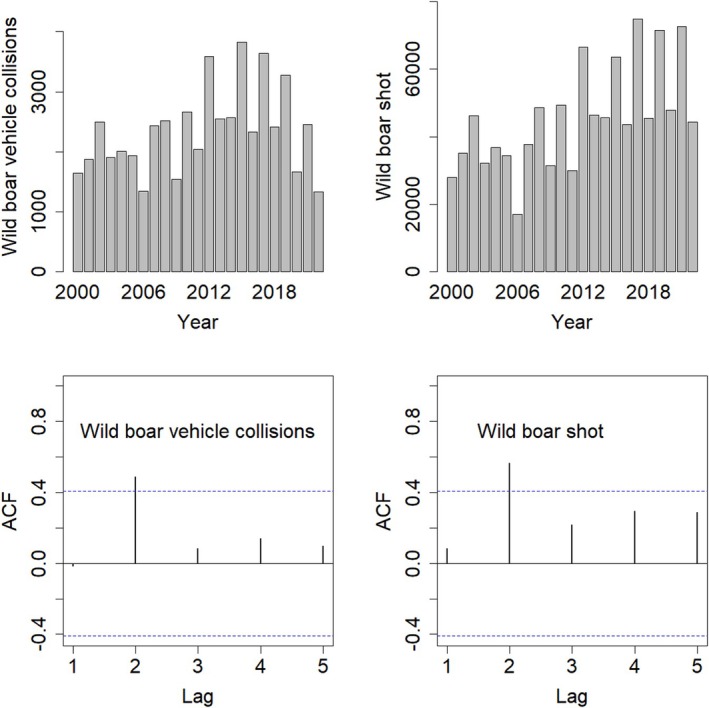
Top: Number of wild boar‐vehicle collisions and wild boar shot (2000–2022). Bottom: Auto correlation function (ACF) for the time series of the number of collisions and the number of wild boar shot (lag period of 1–5 years). Values exceeding those denoted by the horizontal dashed line indicate statistical periodicity.

The number of wild boar‐vehicle collisions neither increased nor decreased since the year 2000 (*p* = 0.15), while during the same period, the number of wild boar shot in Baden‐Württemberg increased (Figure 5, *p*‐value of the linear model using year as covariate = 0.002). The Pearson correlation coefficient between the de‐trended number of wild boar shot and the number of wild boar‐vehicle collisions (2000–2022) was 0.8 (CI_95_ [0.59, 0.91], 2000–2022). The time series of both the number of wild boar shot (2000–2022) and the number of wild boar‐vehicle collisions (2000–2022) were characterized by a significant lag time of 2 years (Figure 5).

**FIGURE 6:**
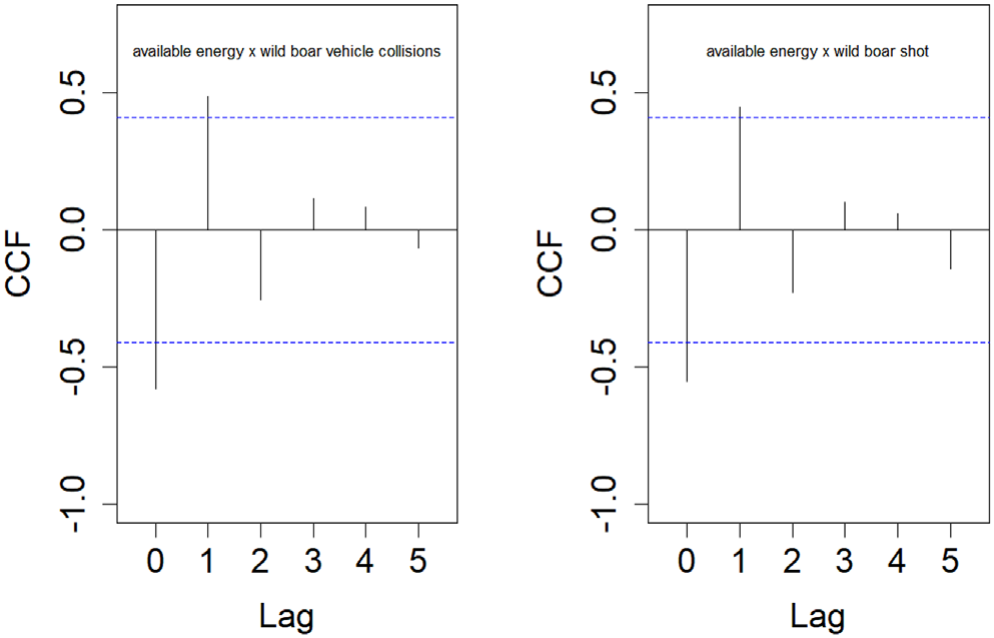
Cross correlation function (CCF) for the time series of available energy and the number of collisions and for the available energy and the number of wild boar shot. Values exceeding those denoted by the horizontal dashed line indicate statistical correlation.

Moreover, the number of shot wild boar and the number of wild boar‐vehicle collisions peaked during years without mast seeding events (Figure 6).

An analysis using the available energy (Equation 1) as the predictor variable and the de‐trended number of wild boar shot or the number of wild boar‐vehicle collisions as the response variable revealed significantly negative associations (2000–2022, adjusted *R* ^2^ = 0.55 and 0.29, respectively, *p* < 0.01). Using the available energy for each tree species (AE_FS_, AE_QP_, AE_QR_—Equation 1) had almost no benefit in explaining the variation of the response variables (wild boar‐vehicle collisions: adjusted *R* ^2^ = 0.36, all *p*‐values > 0.05; de‐trended number of wild boar shot: adjusted *R* ^2^ = 0.63, only the *p*‐value for the available energy related to beech was < 0.05). The temperature during April and May explained 40%, 27%, and 18% of the variation in the available energy related to mast seeding by *Q. petraea* , *Q. robur* , and *F. sylvatica* , respectively. The overall energy of these tree species available for wild boar explained 55% of the annual variation in the number of wild boar shot. Interestingly, the indirect effect (cascading effect) of temperature on the number of wild boar shot annually (temperature during April/May affects the flowering of wind‐pollinated species in the northern hemisphere that affects mast seeding, which in turn affects hunting success), while significant, was relatively small (negative association, adjusted *R* ^2^ = 0.14, *p* = 0.04, 1995–2022).

## 4 Discussion

### 4.1 Wild Boar Bag Records

A bi‐annual pattern of wild boar bag records since 2000 characterized the federal states of southern and western Germany (7 out of 13), neighboring countries (Austria, Czech Republic, Switzerland), as well as Slovenia (2000–2022) and almost all countries of Central Europe (2007–2012 and 2017–2022). A detailed analysis of the data from Baden‐Württemberg (southern Germany) provided insights into the effects of mast seeding on the bag records of wild boar. Specifically, temperature variations during spring (April and May in southern Germany) might be a major driver of mast seeding by *Q. petraea* , *Q. robur* , and *F. sylatica*. During the period 2017–2022, synchronized mast seeding by all three tree species (in 2018, 2020, and 2022) likely increased the population size while reducing the hunting success (Elliger 2025), whereas the number of wild boar shot and the number of wild boar‐vehicle collisions peaked in the intervening years (2019, 2021, and 2023). Thus, years characterized by warm weather conditions during April and May coincided with pulsed resource events and were followed by a year with high bag records. That mast seeding events influence the demographics of wild boar populations has been well‐documented (Cutini et al. 2013; Gamelon et al. 2021, 2019). Wild boar in Central Europe feed on beech and oak seeds during autumn and winter (Vetter et al. 2015). For wild boar, among the positive effects of abundant seedling production by *Quercus* species is an enhanced proportion of breeding females (Touzot et al. 2020) that may outweigh the impact of cold winter weather on wild boar physiology (Vetter et al. 2015). Mast seeding may thus enable wild boar to optimize its theoretical reproductive potential (Apollonio et al. 2010; Cutini et al. 2013; Bieber and Ruf 2005).

Previous work suggested that the bi‐annual pattern of wild boar bag records reflects the success of hunting wild boar using bait (Elliger 2025). In years without mast seeding, wild boar individuals were more likely to be attracted to the presence of bait and thus more likely to be shot by hunters. Furthermore, the absence/presence of pulsed food resources may alter the spatio‐temporal movement behavior of wild boar (Bisi et al. 2018, wild boar home range size was negatively associated with seed biomass production), which would also explain the bi‐annual pattern of wild‐boar‐vehicle collisions.

### 4.2 Mast Seeding

Mast seeding is a complex ecological phenomenon influenced by several factors, including resource supply, weather cues, and fitness benefits (Bogdziewicz et al. 2024; Dale et al. 2021; Koenig 2021). Among the weather cues for wind‐pollinated species in the northern hemisphere is the air temperature during the flowering period (Vacchiano et al. 2017; Caignard et al. 2017). In this study, the temperature during April and May served as a predictor variable for the available energy related to both oak and beech seeds. While this time window may be well suited to approximate year‐to‐year changes in the seed numbers of both temperate species of oak (Caignard et al. 2017; Journé et al. 2021), its predictive power in approximating year‐to‐ year changes in the number of beech seeds might be limited (Vacchiano et al. 2017; Journé et al. 2024). However, the interplay of the various factors resulting in the intra‐ and inter‐specific synchrony of mast seeding differs in different tree species, such that the impacts of water availability, temperature, and other climate variables (Bogdziewicz et al. 2021, 2020; Foest et al. 2025; Kelly et al. 2013) are unclear. Data from *Q. robur* , *Q. petraea* , and *F. sylvatica* in southern Germany indicated synchronized mast seeding between 2017 and 2022.

## 5 Conclusions

In this study, we presented evidence that the bi‐annual pattern observed in the time series of both the number of wild boar shot and the number of wild boar‐vehicle collisions was likely triggered by the mast seeding of beech and oak, at least for the federal state of Baden‐Württemberg (Germany) since 2000. Nonetheless, the temporal pattern in wild boar bag records was the same, not only in other parts of Germany (Figure 1) but also in Austria and Switzerland and, in part, for Poland, Czech‐Republic, Slovak‐ Republic, Slovenia, and France (Figure 3). Although the time series of bag records (harvest data, animal‐vehicle collisions) is not directly linked to species abundance (especially on a local scale), the similarities in the temporal patterns of bag records in Central Europe point to the existence of factors affecting the underlying wild boar population size. In Central Europe, wild boar feed on oak and beech seeds during winter (Vetter et al. 2015; Linderoth 2010). Mast seeding by those tree species, and thus the availability of high‐quality food, has been associated with a higher proportion of breeding females in wild boar (Touzot et al. 2020), resulting in an increased reproductive potential of the population (Cutini et al. 2013, Bieber and Ruf 2005). Moreover, mast seeding can be expected to reduce both the success of hunting wild boar using bait and the home range size (Bisi et al. 2018). The latter likely reduces the chances of collisions between wild boar and vehicles. Both processes were well reflected by the bi‐annual pattern in bag records (Figures 2, 3, 4). The combined effect (collisions, hunting) will likely influence not only the number of wild boar in the year following a mast event but also the demographics of wild boar populations during subsequent years. However, if mast seeding events occur with a frequency of 2 years, then a lag period exceeding the frequency of mast events will be difficult to detect (Figure 6).

### 5.1 Implications for Wildlife Management in Europe

Hagen and co‐authors (Hagen et al. 2017) proposed that the number of ungulate species characterized by more frequent population fluctuations will increase as climate variability increases. Moreover, it is well known that the variance in population size is linked to the mean population size (Taylors law, Taylor 1961). In this study we identified a synchronized spatio‐temporal pattern in wild boar bag records since 2000 in large parts of Europe. However, wild boar bag records in the Baltic region (2014, 2015), Poland and Croatia (since 2021), as well as in Scandinavia (Sweden, Norway, Finland), point to several important factors affecting the population dynamics of free‐ranging large mammals in Europe, namely:

- Range shifts towards the north due to an increase in the mean temperature.
- Irruptive dynamics due to severe viral infections.
- Population fluctuations due to climate change.

## Author Contributions

**Robert Hagen:** conceptualization (equal), data curation (equal). **Christian Fiderer:** conceptualization (equal), data curation (equal), funding acquisition (equal). **Janosch Arnold:** conceptualization (equal), data curation (equal). **Toralf Bauch:** conceptualization (equal), data curation (equal). **Andreas Elliger:** conceptualization (equal), methodology (equal).

## Funding

The authors have nothing to report.

## Conflicts of Interest

The authors declare no conflicts of interest.

## Supporting information


**Figure S1:** Wild boar bag records [1/100 km^2^] for Poland, Czech Republic, Slovak Republic (on the left) and for Germany, Hungary and Luxembourg (on the right).
**Figure S2:** Wild boar bag records [1/100 km^2^] for Latvia, Estonia, Lithuania (on the left) and for France, Spain and Portugal (on the right).
**Figure S3:** Wild boar bag records [1/100 km^2^] for Austria, Switzerland, Slovenia (on the left) and for Croatia, Belgium and Italy (on the right).
**Figure S4:** Wild boar bag records [1/100 km^2^] for Denmark, Norway, Finland (on the left) and for Sweden and Serbia (on the right).
**Figure S5:** ACF for the time series on the number of wild boar bag records for the federal states (Germany) of Baden‐Württemberg, Bavaria and Thuringia (2000–2022).
**Figure S6:** ACF for the time series on the number of wild boar bag records for the federal states (Germany) of North Rhine‐Westphalia, Hesse, Rhineland‐Palatinate and Saarland (2000–2022).
**Figure S7:** ACF for the time series on the number of wild boar bag records for the federal states (Germany) of Brandenburg, Saxony, Saxony‐Anhalt (at the top) and Lower Saxony, Schleswig‐Holstein and Mecklenburg‐Western Pomerania (at the bottom) (2000–2022).
**Figure S8:** ACF for time series on wild boar bag records for Germany, Austria, and Switzerland since 2000.
**Figure S9:** ACF for time series on wild boar bag records from the top to the bottom and from left to right for Czech Republic, Poland, Slovak Republic and Slovenia since 2000.
**Figure S10:** ACF for time series on wild boar bag records for the top to the bottom and from the left to the right for Croatia, France, Spain and Sweden since 2000.
**Figure S11:** ACF for time series on the available energy from the left to the right related to *Quercus Petraea* , *Quercus robur* and *Fagus sylvatica* .
**Figure S12:** ACF of the time series on temperature in April and May (sum) (1995–2022).

**Table S1:** Annual data on wild boar bag records, the area covered with trees older than 80 years (oak) or 60 years (beech), the mast intensity and the estimated energy for metabolism of wild boar for the federal state of Baden‐Württemberg.
**Table S2:** Annual number of wild boar bag records and wild boar vehicle collisions for the federal state of Baden‐Württemberg as well as the mean air temperature in April and May (derived from the German Weather Service—Deutscher Wetterdienst).
**Table S3:** Annual number of wild boar bag records for different federal states in Germany (data were obtained from the German Hunting Association (Deutscher Jagdverband)).
**Table S4:** Annual wild boar bag records for different European countries (2000–2023).

## Data Availability

All data supporting the findings of this study are available within the Supporting Information.
