# Supplementary material for: Pulsed Resource Events Mediate Fluctuations in Wild Boar (Sus scrofa) Bag Records in Central Europe
Source: Ecol Evol. 2026 Jul 1;16(7):e73874. doi: 10.1002/ece3.73874 (PMC13319901; doi:10.1002/ece3.73874)
Supplement: Supplementary file 1 — Figure S1: Wild boar bag records [1/100 km2] for Poland, Czech Republic, Slovak Republic (on the left) and for Germany, Hungary and Luxembourg (on the right). Figure S2: Wild boar bag records [1/100 km2] for Latvia, Estonia, Lithuania (on the left) and for France, Spain and Portugal (on the right). Figure S3: Wild boar bag records [1/100 km2] for Austria, Switzerland, Slovenia (on the left) and for Croatia, Belgium and Italy (on the right). Figure S4: Wild boar bag records [1/100 km2] for Denmark, Norway, Finland (on the left) and for Sweden and Serbia (on the right). Figure S5: ACF for the time series on the number of wild boar bag records for the federal states (Germany) of Baden‐Württemberg, Bavaria and Thuringia (2000–2022). Figure S6: ACF for the time series on the number of wild boar bag records for the federal states (Germany) of North Rhine‐Westphalia, Hesse, Rhineland‐Palatinate and Saarland (2000–2022). Figure S7: ACF for the time series on the number of wild boar bag records for the federal states (Germany) of Brandenburg, Saxony, Saxony‐Anhalt (at the top) and Lower Saxony, Schleswig‐Holstein and Mecklenburg‐Western Pomerania (at the bottom) (2000–2022). Figure S8: ACF for time series on wild boar bag records for Germany, Austria, and Switzerland since 2000. Figure S9: ACF for time series on wild boar bag records from the top to the bottom and from left to right for Czech Republic, Poland, Slovak Republic and Slovenia since 2000. Figure S10: ACF for time series on wild boar bag records for the top to the bottom and from the left to the right for Croatia, France, Spain and Sweden since 2000. Figure S11: ACF for time series on the available energy from the left to the right related to Quercus Petraea , Quercus robur and Fagus sylvatica . Figure S12: ACF of the time series on temperature in April and May (sum) (1995–2022). [file ECE3-16-e73874-s002.docx]

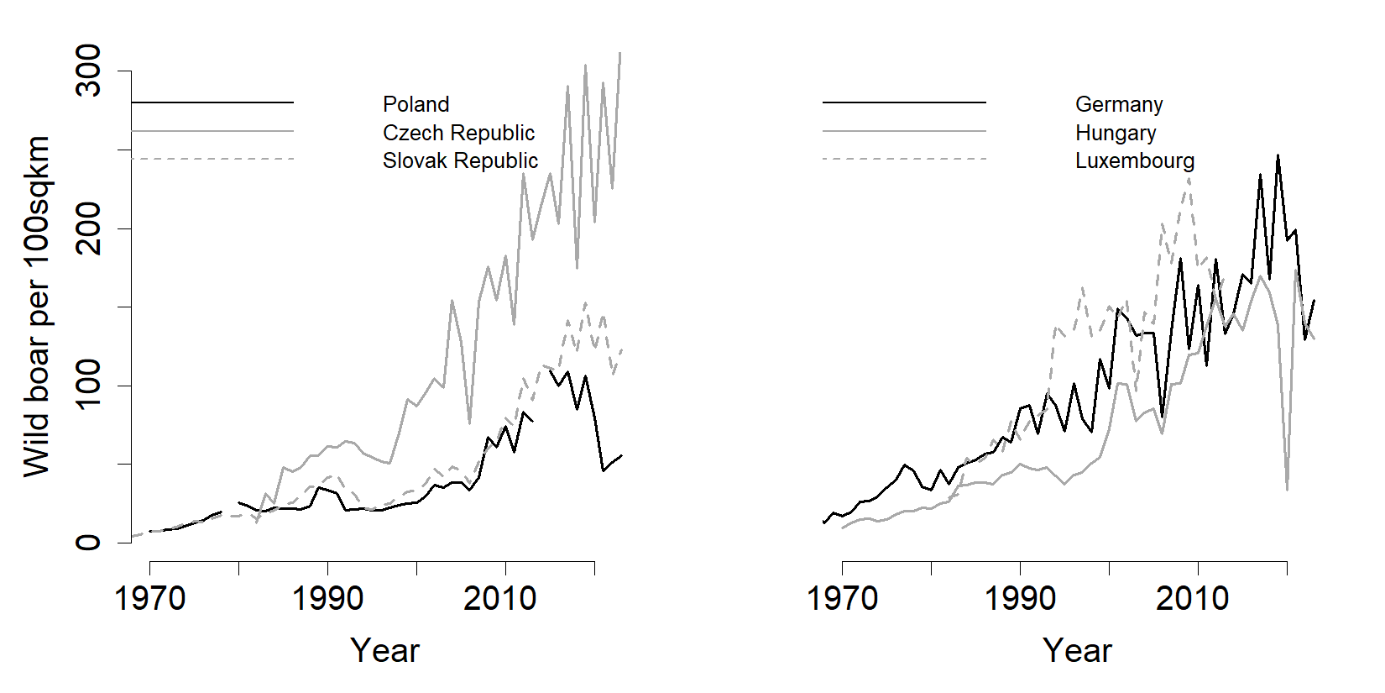


*Fig S1 Wild boar bag records [1/ 100km^2^] for Poland, Czech Republic, Slovak Republic (on the left) and for Germany, Hungary and Luxembourg (on the right)*


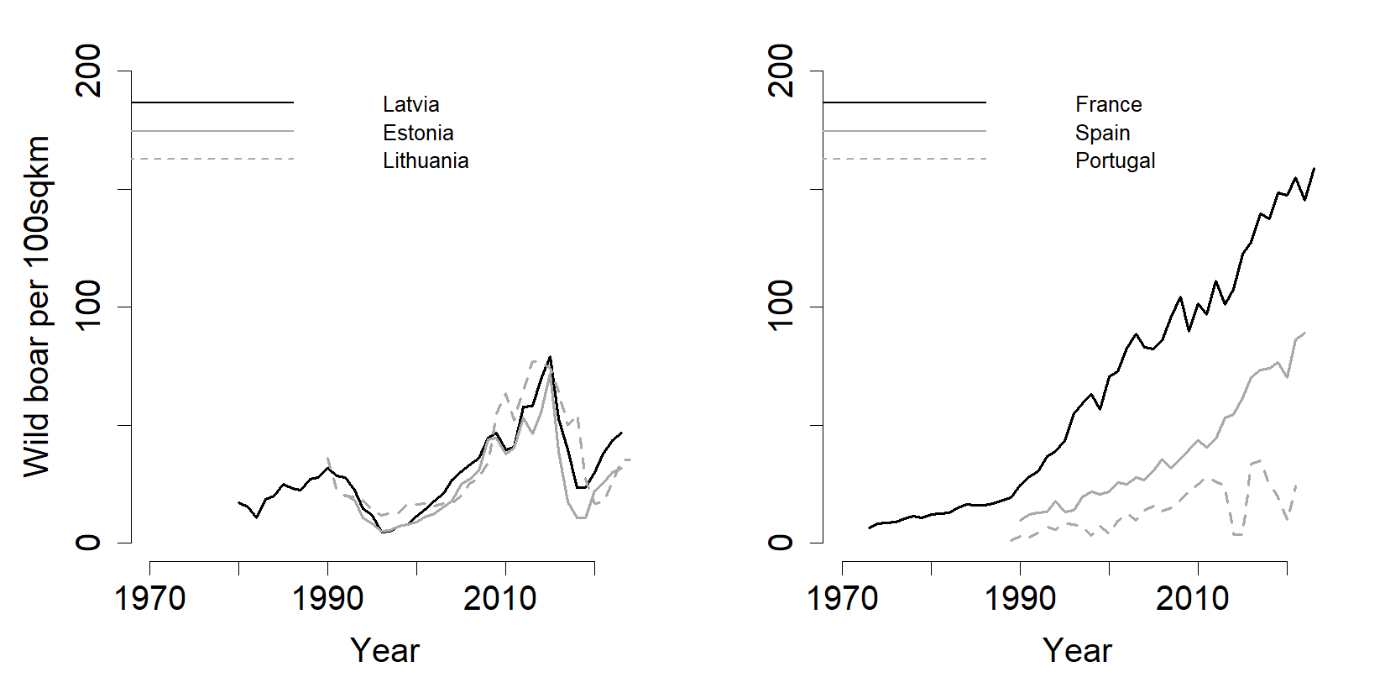


*Fig S2 Wild boar bag records [1/ 100km^2^] for Latvia, Estonia, Lithuania (on the left) and for France, Spain and Portugal (on the right)*


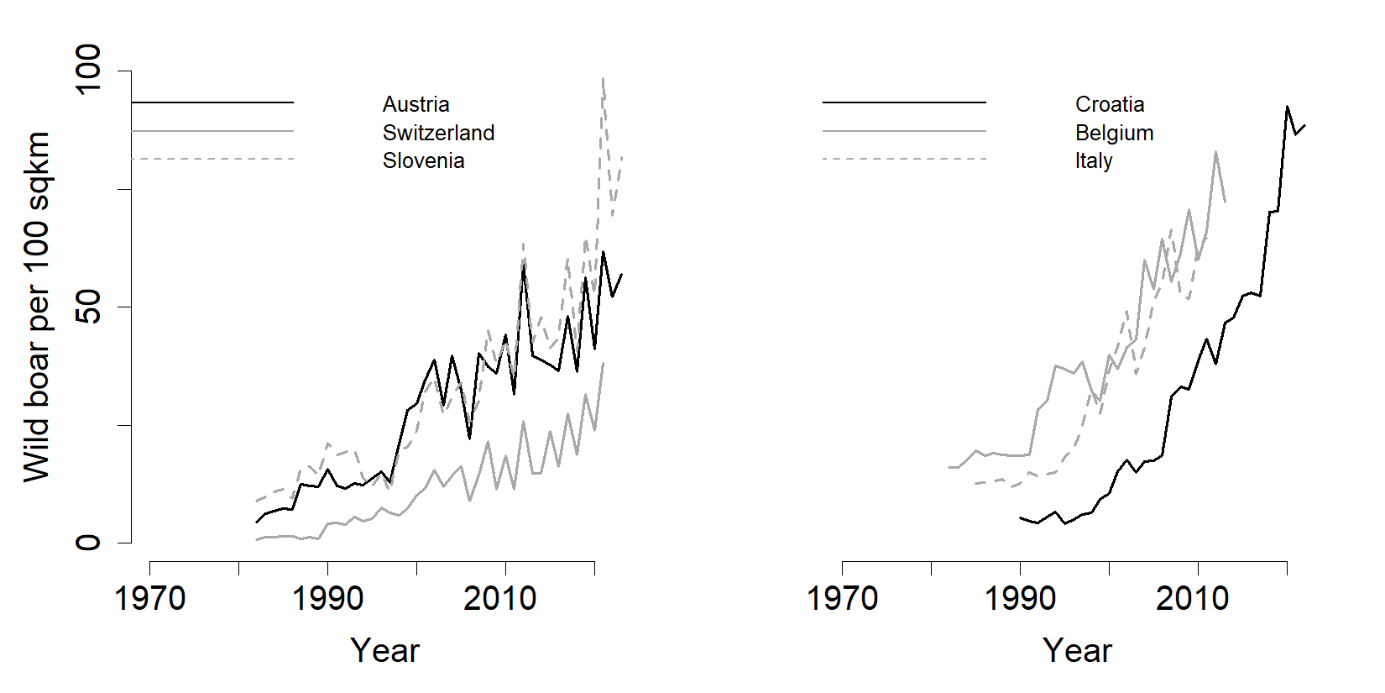


*Fig S3 Wild boar bag records [1/ 100km^2^] for Austria, Switzerland, Slovenia (on the left) and for Croatia, Belgium and Italy (on the right)*


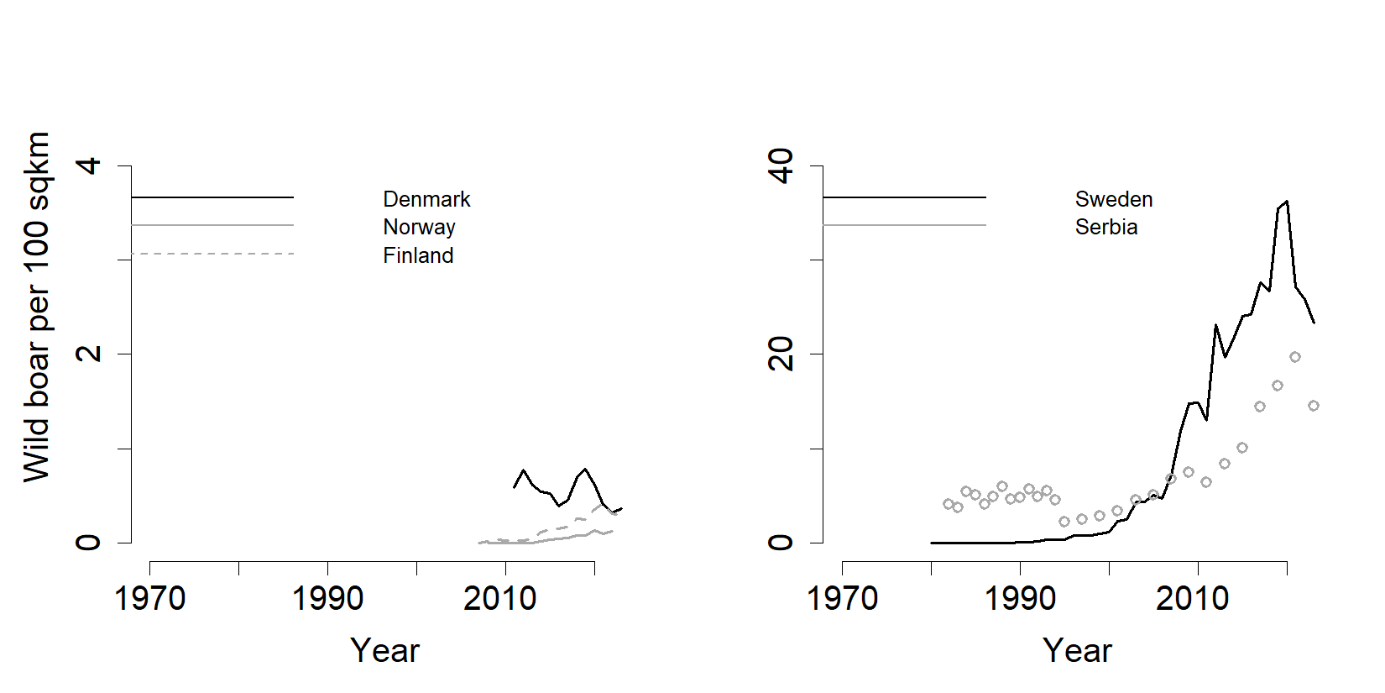


*Fig S4 Wild boar bag records [1/ 100km^2^] for Denmark, Norway, Finland (on the left) and for Sweden and Serbia (on the right)*

*
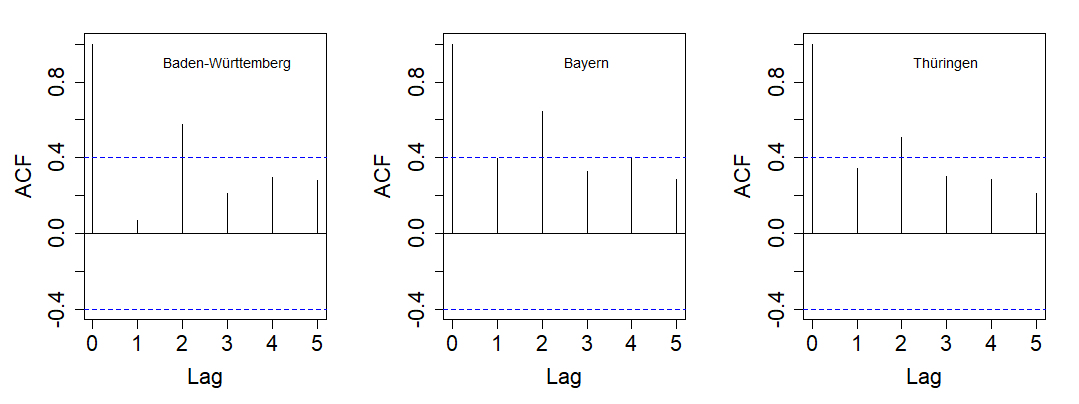
*

*Fig S5 ACF for the time series on the number of wild boar bag records for the federal states (Germany) of Baden-Württemberg, Bavaria and Thuringia (2000-2022)*

*
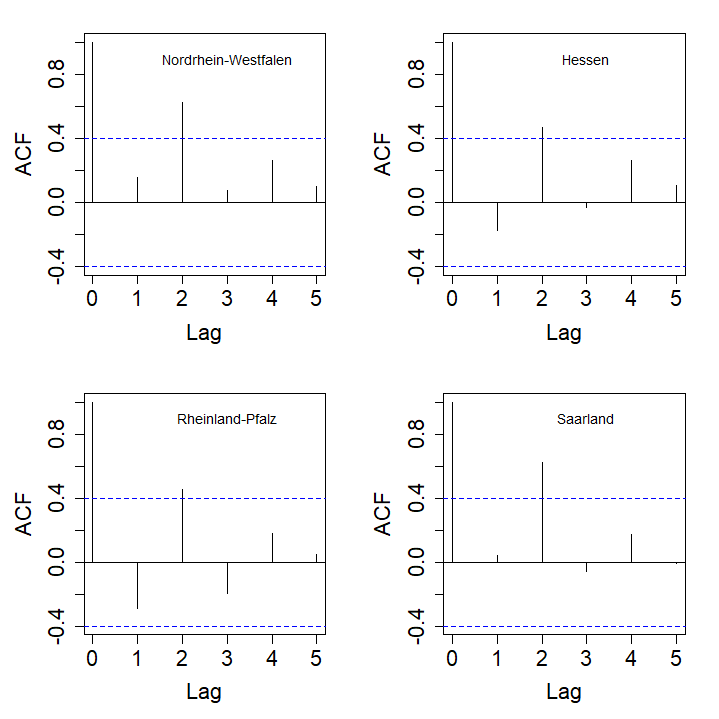
*

*Fig S6 ACF for the time series on the number of wild boar bag records for the federal states (Germany) of North Rhine-Westphalia, Hesse, Rhineland-Palatinate and Saarland (2000-2022)*

*
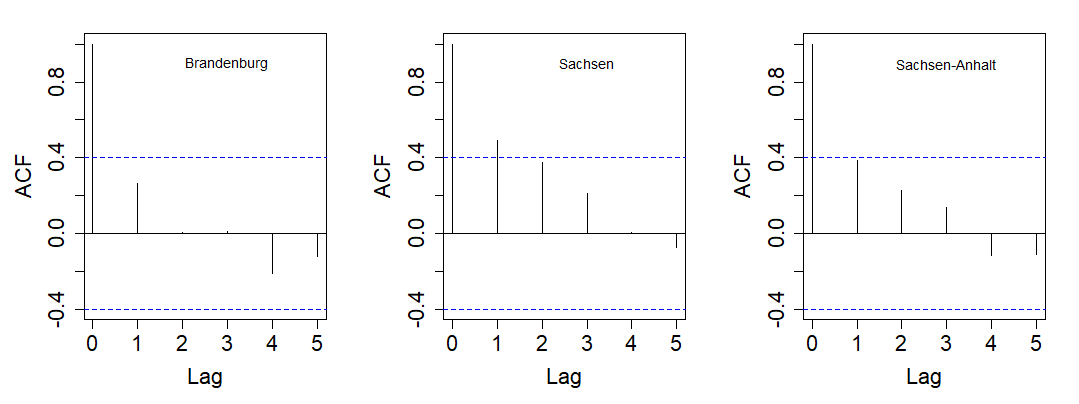
*

*
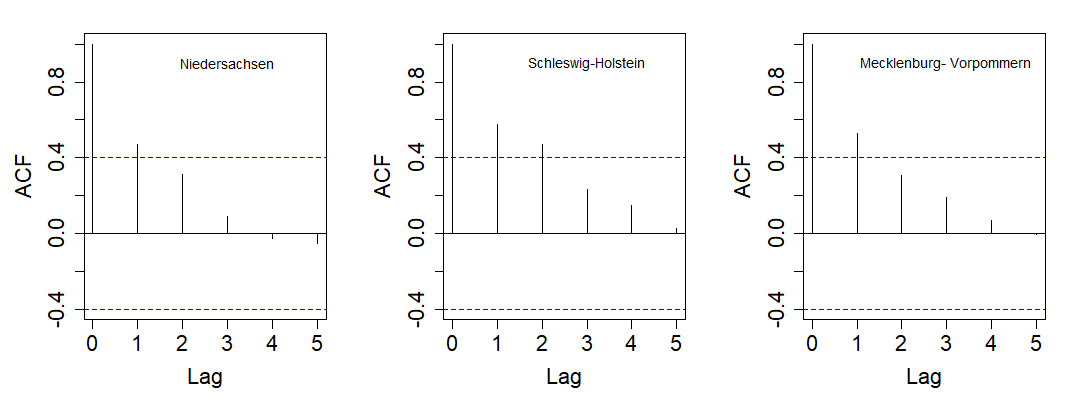
*

*Fig S7 ACF for the time series on the number of wild boar bag records for the federal states (Germany) of Brandenburg, Saxony, Saxony-Anhalt (at the top) and Lower Saxony, Schleswig-Holstein and Mecklenburg-Western Pomerania (at the bottom) (2000-2022)*


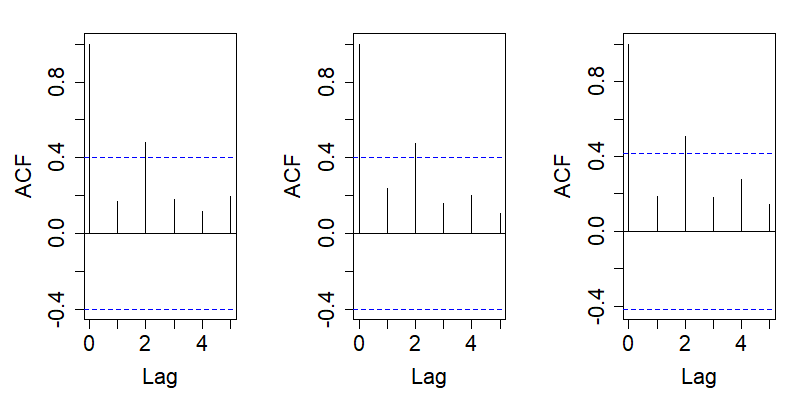
Fig S8 *ACF for time series on wild boar bag records for Germany, Austria, and Switzerland since 2000*
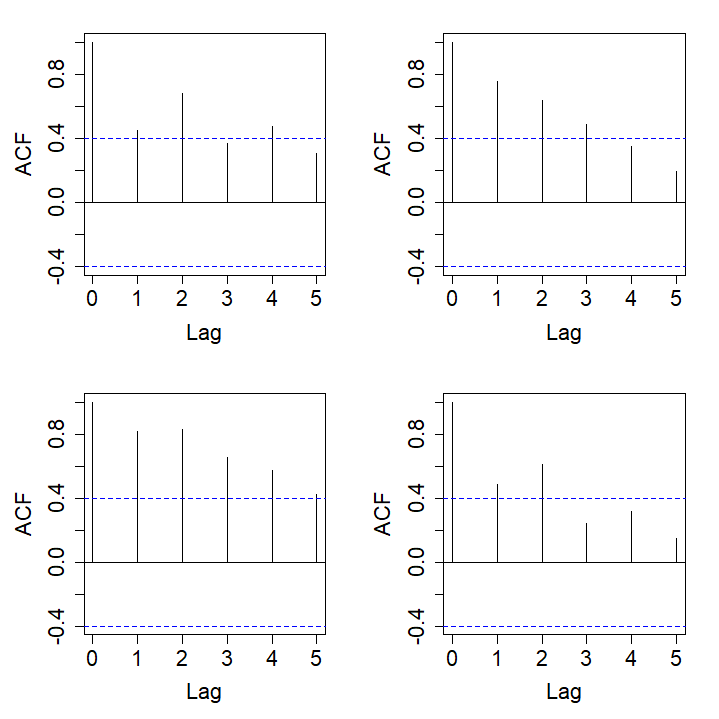


Fig S9 *ACF for time series on wild boar bag records from the top to the bottom and from left to right for Czech Republic, Poland, Slovak Republic and Slovenia since 2000*


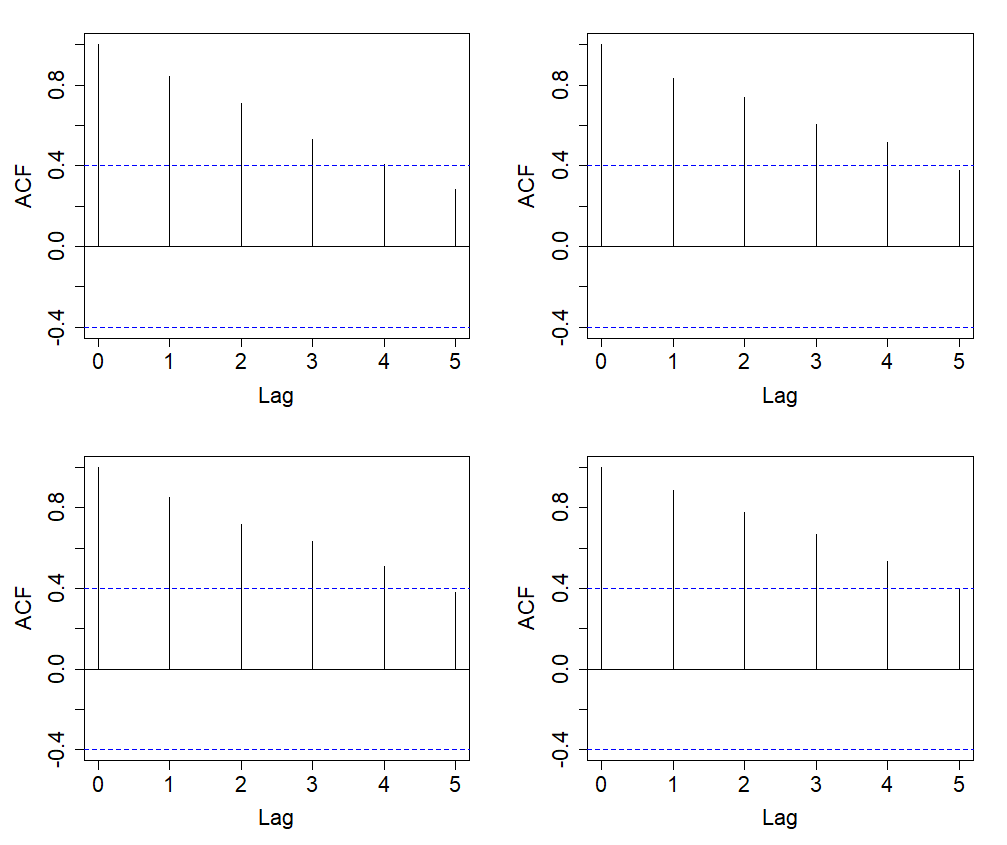


Fig S10 *ACF for time series on wild boar bag records for the top to the bottom and from the left to the right for Croatia, France, Spain and Sweden since 2000*


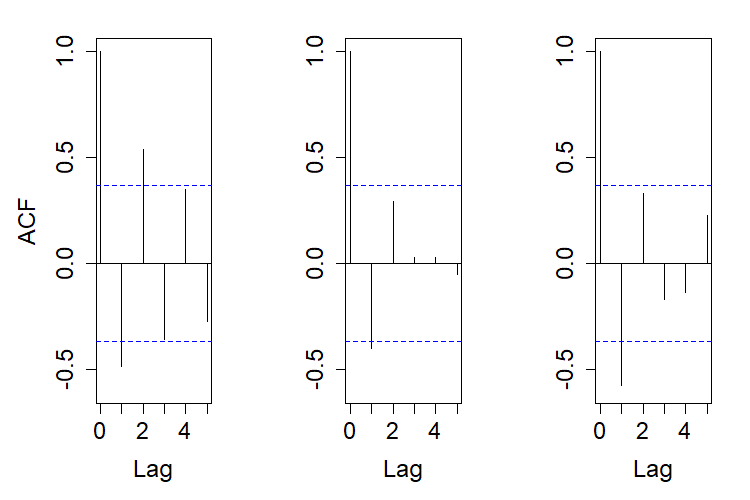


Fig S11 *ACF for time series on the available energy from the left to the right related to Quercus Petraea, Quercus Robur and Fagus Sylvatica*


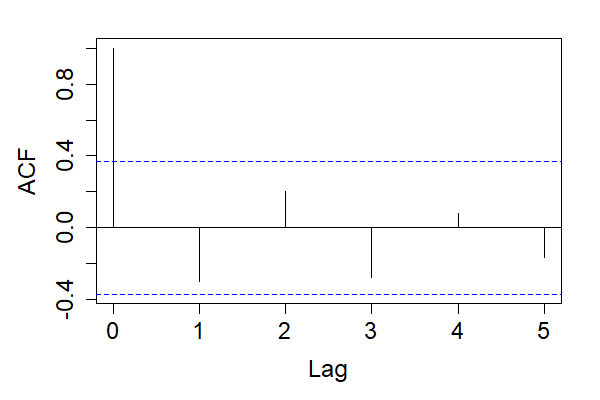


Fig S12 – *ACF of the time series on temperature in April and May (sum) (1995-2022)*
