# Supplementary material for: Pulsed Resource Events Mediate Fluctuations in Wild Boar (Sus scrofa) Bag Records in Central Europe
Source: Ecol Evol. 2026 Jul 1;16(7):e73874. doi: 10.1002/ece3.73874 (PMC13319901; doi:10.1002/ece3.73874)
Supplement: Supplementary file 2 — Table S1: Annual data on wild boar bag records, the area covered with trees older than 80 years (oak) or 60 years (beech), the mast intensity and the estimated energy for metabolism of wild boar for the federal state of Baden‐Württemberg. Table S2: Annual number of wild boar bag records and wild boar vehicle collisions for the federal state of Baden‐Württemberg as well as the mean air temperature in April and May (derived from the German Weather Service—Deutscher Wetterdienst). Table S3: Annual number of wild boar bag records for different federal states in Germany (data were obtained from the German Hunting Association (Deutscher Jagdverband)). Table S4: Annual wild boar bag records for different European countries (2000–2023). [file ECE3-16-e73874-s001.docx]

Tab S1 *Annual data on wild boar bag records, the area covered with trees older than 80 years (oak) or 60 years (beech), the mast intensity and the estimated energy for metabolism of wild boar for the federal state of Baden-Württemberg*.

| Year | Wild boar  bag records (number) | Quercus petraea  (ha) | Quercus  robur  (ha) | Fagus  sylvatica  (ha) | Mast intensity  (Q. petraea) | Mast intensity (Q. robur) | Mast intensity  (F. sylvatica) | Energy  (F. sylvatica) (MJ) | Energy  (Q. robur) (MJ) | Energy  (Q. petraea)  (MJ) | Available Energy (MJ) |
| --- | --- | --- | --- | --- | --- | --- | --- | --- | --- | --- | --- |
| 1995 | 21571 | 23400 | 21030 | 160570 | 0,65 | 0,3 | 0,65 | 1153294 | 34700 | 83655 | 1271649 |
| 1996 | 30398 | 23400 | 21030 | 160570 | 0,30 | 0,3 | 0,05 | 88715 | 34700 | 38610 | 162024 |
| 1997 | 21539 | 23400 | 21030 | 160570 | 0,30 | 0,3 | 0,05 | 88715 | 34700 | 38610 | 162024 |
| 1998 | 21949 | 23400 | 21030 | 160570 | 0,30 | 0,65 | 0,30 | 532290 | 75182 | 38610 | 646082 |
| 1999 | 25782 | 23400 | 21030 | 160570 | 0,30 | 0,05 | 0,65 | 1153294 | 5783 | 38610 | 1197687 |
| 2000 | 29576 | 23400 | 21030 | 160570 | 0,65 | 0,65 | 0,30 | 532290 | 75182 | 83655 | 691127 |
| 2001 | 36940 | 23400 | 21030 | 160570 | 0,65 | 0,65 | 0,65 | 1153294 | 75182 | 83655 | 1312131 |
| 2002 | 48746 | 29240 | 27130 | 196530 | 0,30 | 0,05 | 0,30 | 651497 | 7461 | 48246 | 707204 |
| 2003 | 34146 | 29240 | 27130 | 196530 | 0,90 | 0,9 | 0,30 | 651497 | 134294 | 144738 | 930528 |
| 2004 | 38735 | 29240 | 27130 | 196530 | 0,65 | 0,3 | 0,30 | 651497 | 44765 | 104533 | 800794 |
| 2005 | 36328 | 29240 | 27130 | 196530 | 0,65 | 0,3 | 0,05 | 108583 | 44765 | 104533 | 257880 |
| 2006 | 18377 | 29240 | 27130 | 196530 | 0,30 | 0,65 | 0,65 | 1411577 | 96990 | 48246 | 1556812 |
| 2007 | 40173 | 29240 | 27130 | 196530 | 0,65 | 0,65 | 0,30 | 651497 | 96990 | 104533 | 853020 |
| 2008 | 51086 | 29240 | 27130 | 196530 | 0,65 | 0,05 | 0,05 | 108583 | 7461 | 104533 | 220577 |
| 2009 | 32969 | 29240 | 27130 | 196530 | 0,65 | 0,3 | 0,90 | 1954491 | 44765 | 104533 | 2103788 |
| 2010 | 51931 | 29240 | 27130 | 196530 | 0,30 | 0,05 | 0,05 | 108583 | 7461 | 48246 | 164290 |
| 2011 | 32051 | 29240 | 27130 | 196530 | 0,90 | 0,3 | 0,90 | 1954491 | 44765 | 144738 | 2143993 |
| 2012 | 70147 | 30200 | 28060 | 202840 | 0,65 | 0,05 | 0,05 | 112069 | 7717 | 107965 | 227751 |
| 2013 | 49036 | 30200 | 28060 | 202840 | 0,30 | 0,3 | 0,30 | 672415 | 46299 | 49830 | 768544 |
| 2014 | 48154 | 30200 | 28060 | 202840 | 0,65 | 0,3 | 0,30 | 672415 | 46299 | 107965 | 826679 |
| 2015 | 67490 | 30200 | 28060 | 202840 | 0,05 | 0,3 | 0,05 | 112069 | 46299 | 8305 | 166673 |
| 2016 | 45911 | 30200 | 28060 | 202840 | 0,65 | 0,05 | 0,90 | 2017244 | 7717 | 107965 | 2132925 |
| 2017 | 78551 | 30200 | 28060 | 202840 | 0,05 | 0,3 | 0,05 | 112069 | 46299 | 8305 | 166673 |
| 2018 | 47837 | 30200 | 28060 | 202840 | 0,90 | 0,9 | 0,65 | 1456898 | 138897 | 149490 | 1745285 |
| 2019 | 74734 | 30200 | 28060 | 202840 | 0,05 | 0,05 | 0,30 | 672415 | 7717 | 8305 | 688436 |
| 2020 | 49463 | 30200 | 28060 | 202840 | 0,90 | 0,9 | 0,65 | 1456898 | 138897 | 149490 | 1745285 |
| 2021 | 75098 | 30200 | 28060 | 202840 | 0,05 | 0,05 | 0,05 | 112069 | 7717 | 8305 | 128091 |
| 2022 | 45667 | 36260 | 34830 | 224450 | 0,65 | 0,65 | 0,30 | 744052 | 124517 | 129630 | 998199 |

Tab S2 *Annual number of wild boar bag records and wild boar vehicle collisions for the federal state of Baden-Württemberg as well as the mean air temperature in April and May (derived from the German Weather Service – Deutscher Wetterdienst)*

| Year | Wild boar  bag records (number) | Wild boar vehicle collisions (number) | Temperature April (°C) | Temperature May (°C) | Sum (April + May)  (°C) |
| --- | --- | --- | --- | --- | --- |
| 1995 | 21571 |  | 12,23 | 8,64 | 20,87 |
| 1996 | 30398 |  | 11,4 | 8,35 | 19,75 |
| 1997 | 21539 |  | 12,77 | 6,63 | 19,4 |
| 1998 | 21949 |  | 13,92 | 8,25 | 22,17 |
| 1999 | 25782 |  | 14,32 | 8,53 | 22,85 |
| 2000 | 29576 | 1640 | 14,3 | 9,69 | 23,99 |
| 2001 | 36940 | 1868 | 14,94 | 6,62 | 21,56 |
| 2002 | 48746 | 2498 | 12,74 | 8,15 | 20,89 |
| 2003 | 34146 | 1900 | 14,05 | 8,49 | 22,54 |
| 2004 | 38735 | 2010 | 11,27 | 9,02 | 20,29 |
| 2005 | 36328 | 1938 | 12,85 | 9,01 | 21,86 |
| 2006 | 18377 | 1337 | 12,95 | 7,93 | 20,88 |
| 2007 | 40173 | 2430 | 14,19 | 12,21 | 26,4 |
| 2008 | 51086 | 2519 | 14,82 | 7,4 | 22,22 |
| 2009 | 32969 | 1536 | 14,25 | 11,18 | 25,43 |
| 2010 | 51931 | 2658 | 10,65 | 8,79 | 19,44 |
| 2011 | 32051 | 2036 | 14,15 | 11,42 | 25,57 |
| 2012 | 70147 | 3584 | 14,16 | 8,11 | 22,27 |
| 2013 | 49036 | 2547 | 10,64 | 8,36 | 19 |
| 2014 | 48154 | 2572 | 12,19 | 10,46 | 22,65 |
| 2015 | 67490 | 3822 | 13,09 | 8,83 | 21,92 |
| 2016 | 45911 | 2325 | 12,58 | 7,82 | 20,4 |
| 2017 | 78551 | 3640 | 13,93 | 7,47 | 21,4 |
| 2018 | 47837 | 2416 | 15,23 | 12,5 | 27,73 |
| 2019 | 74734 | 3272 | 10,44 | 9 | 19,44 |
| 2020 | 49463 | 1660 | 12,37 | 11,16 | 23,53 |
| 2021 | 75098 | 2457 | 10,13 | 6,19 | 16,32 |
| 2022 | 45667 | 1327 | 14,88 | 7,85 | 22,73 |
| 2023 | 49847 | 1380 | 13,33 | 7,54 | 20,87 |

*Tab S3 Annual number of wild boar bag records for different federal states in Germany (data were obtained from the German Hunting Association (Deutscher Jagdverband))*

| *Year* | *Schleswig-Holstein* | Lower Saxony | Mecklenburg-Western Pomerania | *Brandenburg* | *Saxony-Anhalt* | *Thuringia* | *Saxony* | North Rhine-Westphalia | *Hesse* | Rhineland-Palatinate | *Saarland* | *Bavaria* |
| --- | --- | --- | --- | --- | --- | --- | --- | --- | --- | --- | --- | --- |
| *2000* | *5756* | *23198* | *33378* | *55274* | *25444* | *32044* | *26336* | *16709* | *31185* | *38333* | *2560* | *27643* |
| *2001* | *9185* | *39581* | *47138* | *66051* | *32891* | *24183* | *33922* | *29612* | *73347* | *58528* | *5274* | *46162* |
| *2002* | *7802* | *44171* | *55371* | *79036* | *36128* | *24183* | *32833* | *21062* | *48218* | *52493* | *4232* | *55265* |
| *2003* | *11248* | *38716* | *52661* | *52596* | *28817* | *22809* | *20787* | *29401* | *61481* | *66665* | *7453* | *41848* |
| *2004* | *8388* | *37144* | *50760* | *73640* | *33092* | *21445* | *29378* | *20223* | *50273* | *51686* | *4317* | *54769* |
| *2005* | *8205* | *37655* | *51075* | *66758* | *30989* | *24561* | *22543* | *29764* | *56874* | *61812* | *6055* | *42085* |
| *2006* | *8170* | *26514* | *38777* | *43359* | *17040* | *12709* | *13269* | *16697* | *31947* | *29001* | *3671* | *26246* |
| *2007* | *11576* | *49760* | *58155* | *64500* | *29826* | *22046* | *24009* | *30469* | *50098* | *43589* | *4858* | *48637* |
| *2008* | *14541* | *57604* | *75866* | *80151* | *35647* | *29926* | *28649* | *42869* | *77927* | *80175* | *6483* | *62195* |
| *2009* | *14401* | *50080* | *57843* | *60640* | *28749* | *20276* | *24416* | *21518* | *41843* | *39223* | *4196* | *42545* |
| *2010* | *16092* | *55295* | *64044* | *72505* | *33631* | *27271* | *26468* | *34224* | *68107* | *65576* | *6864* | *60533* |
| *2011* | *9203* | *38580* | *47320* | *60847* | *26801* | *21141* | *22575* | *21138* | *41050* | *35335* | *3016* | *42312* |
| *2012* | *14743* | *49881* | *65059* | *71837* | *34573* | *37422* | *32201* | *40076* | *74728* | *79228* | *6876* | *65718* |
| *2013* | *9155* | *39015* | *47682* | *63254* | *27893* | *23468* | *26173* | *22350* | *52481* | *40359* | *3467* | *68679* |
| *2014* | *11273* | *42104* | *55464* | *70857* | *29551* | *25831* | *27710* | *30170* | *54356* | *48134* | *3842* | *71203* |
| *2015* | *12556* | *44936* | *57951* | *70916* | *33862* | *31232* | *32878* | *34447* | *69507* | *61847* | *5872* | *85436* |
| *2016* | *15694* | *56185* | *60764* | *76512* | *39298* | *31052* | *33259* | *38954* | *62365* | *60722* | *5728* | *60875* |
| *2017* | *19503* | *68992* | *85949* | *89819* | *49219* | *41897* | *45318* | *66079* | *96001* | *88650* | *8814* | *95096* |
| *2018* | *16276* | *55742* | *73177* | *71455* | *38125* | *29458* | *36087* | *39427* | *61549* | *57143* | *6831* | *65455* |
| *2019* | *19864* | *70481* | *96559* | *102456* | *46148* | *45814* | *47061* | *64736* | *84375* | *100072* | *13128* | *114844* |
| *2020* | *21286* | *61960* | *106803* | *90306* | *39733* | *38556* | *37069* | *34251* | *65351* | *55230* | *6631* | *78064* |
| *2021* | *15801* | *57229* | *77212* | *58296* | *32430* | *35358* | *32369* | *49566* | *88378* | *82747* | *10881* | *93526* |
| *2022* | *10535* | *36134* | *49831* | *45550* | *26527* | *27216* | *22004* | *29991* | *53841* | *45107* | *5049* | *63152* |
| *2023* | *13988* | *42499* | *66036* | *46600* | *29650* | *30695* | *23729* | *41611* | *63925* | *61820* | *7227* | *71653* |

*Tab S4 Annual wild boar bag records for different European countries (2000-2023).* Data were obtained from numerous institutions (cf. Table 1 – main text).

| Year | Sweden | Latvia | Slovakia | Slovenia | Czech republic | Germany | Poland | Hungary | Austria | Switzerland | Portugal | Croatia | France | Spain | Lithuania | Estonia |
| --- | --- | --- | --- | --- | --- | --- | --- | --- | --- | --- | --- | --- | --- | --- | --- | --- |
| 2000 | 5211 | 7404 | 16448 | 4800 | 68472 | 350976 | 81000 | 67700 | 24822 | 4160 | 3617 | 5986 | 383943 | 110000 | 10810 | 3952 |
| 2001 | 10440 | 9295 | 18236 | 6500 | 74832 | 531887 | 92000 | 94500 | 28926 | 4787 | 8669 | 8537 | 394956 | 130000 | 10883 | 4937 |
| 2002 | 11375 | 11511 | 23161 | 7103 | 82536 | 512050 | 115000 | 94000 | 32494 | 6376 | 11576 | 9971 | 449466 | 125000 | 10300 | 5660 |
| 2003 | 19616 | 13629 | 20610 | 5472 | 77871 | 470283 | 110000 | 72100 | 24520 | 4943 | 8809 | 8452 | 482713 | 140000 | 10843 | 7003 |
| 2004 | 19735 | 17161 | 23727 | 6292 | 121956 | 476042 | 120000 | 77200 | 33370 | 5911 | 12872 | 9803 | 450578 | 135000 | 10919 | 8122 |
| 2005 | 22682 | 19516 | 22551 | 6892 | 100557 | 476645 | 120000 | 79500 | 27223 | 6696 | 14323 | 9827 | 447597 | 155000 | 13022 | 11332 |
| 2006 | 21170 | 21504 | 18483 | 5204 | 59868 | 287080 | 105000 | 64400 | 18540 | 3662 | 12548 | 10445 | 466352 | 180000 | 16554 | 12225 |
| 2007 | 32628 | 23247 | 25758 | 6114 | 121020 | 479907 | 130000 | 94000 | 33778 | 5951 | 13869 | 17527 | 523331 | 160000 | 17800 | 13818 |
| 2008 | 53236 | 28862 | 29700 | 9132 | 138723 | 646790 | 210000 | 94400 | 31320 | 8866 | 16826 | 18679 | 568355 | 180000 | 22366 | 19757 |
| 2009 | 66625 | 30201 | 31473 | 7721 | 121690 | 440354 | 190000 | 111200 | 30212 | 4685 | 20102 | 18409 | 488830 | 200000 | 35814 | 20072 |
| 2010 | 66781 | 25568 | 38903 | 8742 | 144184 | 585244 | 232700 | 112400 | 37115 | 7647 | 23043 | 21871 | 551141 | 220000 | 41441 | 17028 |
| 2011 | 58602 | 26332 | 36390 | 7110 | 109383 | 402507 | 180000 | 128900 | 26380 | 4726 | 25608 | 24496 | 526904 | 205000 | 33922 | 18159 |
| 2012 | 104303 | 37288 | 51296 | 12838 | 185176 | 644239 | 260000 | 144700 | 49734 | 10653 | 24082 | 21436 | 604720 | 222692 | 42263 | 24080 |
| 2013 | 88476 | 37576 | 44549 | 8607 | 152250 | 474363 | 242000 | 128400 | 33277 | 6129 | 22233 | 26394 | 550619 | 268655 | 50172 | 20885 |
| 2014 | 97330 | 44871 | 55661 | 9703 | 168974 | 520623 |  | 135800 | 32559 | 6067 | 3356 | 26997 | 585587 | 274728 | 50172 | 24909 |
| 2015 | 108013 | 50956 | 54401 | 8367 | 185496 | 610631 | 342100 | 125600 | 31669 | 9799 | 3179 | 29563 | 666933 | 310280 | 48317 | 32580 |
| 2016 | 108891 | 34084 | 53788 | 8822 | 160139 | 589417 | 311700 | 143100 | 30594 | 6673 | 30916 | 30000 | 693613 | 354648 | 42188 | 17610 |
| 2017 | 124192 | 25549 | 69509 | 12238 | 229182 | 836865 | 341411 | 158100 | 40297 | 11346 | 32126 | 29599 | 758711 | 370770 | 32624 | 7960 |
| 2018 | 120229 | 15238 | 59253 | 8250 | 137823 | 599855 | 266000 | 148000 | 30542 | 7727 | 23014 | 39713 | 747568 | 373225 | 35577 | 4761 |
| 2019 | 159384 | 15279 | 74947 | 13176 | 239818 | 882231 | 331900 | 128500 | 47251 | 12996 | 18003 | 39778 | 807753 | 385726 | 18016 | 4820 |
| 2020 | 163140 | 19262 | 59656 | 10645 | 160811 | 687581 | 249100 | 31500 | 34541 | 9819 | 8909 | 52381 | 801513 | 354374 | 10935 | 9978 |
| 2021 | 122149 | 24485 | 72116 | 19927 | 230905 | 711407 | 143800 | 161300 | 51758 | 15728 | 22414 | 48893 | 842802 | 434542 | 11264 | 11401 |
| 2022 | 116168 | 28006 | 52163 | 14052 | 177877 | 462220 | 160500 | 130200 | 43814 |  |  | 50030 | 789816 | 450150 | 16885 | 13525 |
| 2023 | 105010 | 30153 | 60232 | 16536 | 258253 | 550510 | 173700 | 121000 | 47821 |  |  |  | 863124 |  | 22980 | 14293 |
